# Key Therapeutic Targets to Treat Hyperglycemia-Induced Atherosclerosis Analyzed Using a Petri Net-Based Model

**DOI:** 10.3390/metabo13121191

**Published:** 2023-12-08

**Authors:** Agnieszka Rybarczyk, Dorota Formanowicz, Piotr Formanowicz

**Affiliations:** 1 Institute of Computing Science, Poznan University of Technology, 60-695 Poznan, Poland; Agnieszka.Rybarczyk@cs.put.poznan.pl; 2 Institute of Bioorganic Chemistry, Polish Academy of Sciences, 61-704 Poznan, Poland; 3 Faculty of Electrical Engineering, Gdynia Maritime University, 81-225 Gdynia, Poland; 4 Department of Medical Chemistry and Laboratory Medicine, Poznan University of Medical Sciences, 60-806 Poznan, Poland; doforman@ump.edu.pl

**Keywords:** diabetes mellitus, atherosclerosis, modeling, Petri nets, knockout analysis

## Abstract

Chronic superphysiological glucose concentration is a hallmark of diabetes mellitus (DM) and a cause of damage to many types of cells. Atherosclerosis coexists with glucose metabolism disturbances, constituting a significant problem and exacerbating its complications. Atherosclerosis in DM is accelerated, so it is vital to slow its progression. However, from the complex network of interdependencies, molecules, and processes involved, choosing which ones should be inhibited without blocking the pathways crucial for the organism’s functioning is challenging. To conduct this type of analysis, in silicotesting comes in handy. In our study, to identify sites in the network that need to be blocked to have an inhibitory effect on atherosclerosis in hyperglycemia, which is toxic for the human organism, we created a model using Petri net theory and performed analyses. We have found that blocking isoforms of protein kinase C (PKC)—PKCβ and PKCγ—in diabetic patients can contribute to the inhibition of atherosclerosis progression. In addition, we have discovered that aldose reductase inhibition can slow down atherosclerosis progression, and this has been shown to reduce PKC (β and γ) expression in DM. It has also been observed that diminishing oxidative stress through the inhibitory effect on the AGE-RAGE axis may be a promising therapeutic approach in treating hyperglycemia-induced atherosclerosis. Moreover, the blockade of NADPH oxidase, the key enzyme responsible for the formation of reactive oxygen species (ROS) in blood vessels, only moderately slowed down atherosclerosis development. However, unlike aldose reductase blockade, or direct PKC (β and γ), the increased production of mitochondrial ROS associated with mitochondrial dysfunction effectively stopped after NADPH oxidase blockade. The results obtained may constitute the basis for further in-depth research.

## 1. Introduction

### 1.1. Research Context

Glucose metabolism disturbances, mainly diabetes mellitus (DM), affect people worldwide. DM refers to a group of metabolic disorders caused by impaired insulin secretion, malfunction of its action, or both, leading to hyperglycemia.

Insulin is a vital human metabolism hormone; hence, its secretion is highly regulated, and its deficiency or malfunction leads to many metabolic disorders affecting not only carbohydrate but also protein and lipid metabolic pathways [1,2,3].

Disturbances in glucose homeostasis lead to a chronic increase in blood glucose concentrations, adversely affecting many organs and tissues. As a life-threatening risk factor, hyperglycemia-induced chronic glucotoxicity causes damage (glucose toxicity) to many types of cells through both chronic and acute processes. It is strongly correlated with numerous complications associated with long-term damage, dysfunction, and organ failure, especially of the kidneys, eyes, and nervous and cardiovascular systems, and accelerates the development of atherosclerosis. DM patients’ most common cardiovascular manifestations include peripheral arterial disease and ischemic heart disease, which leads to heart failure. Although DM predisposes patients to cardiovascular disease (CVD), it is not actually equivalent to risk but is associated with significant CVD risk heterogeneity. The complexity of this problem is high, resulting from altered lipid metabolism, oxidative stress, and low-grade local inflammation that coexist with DM and influence each other [4]. Extensive research has thoroughly investigated the link between DM and atherosclerosis, revealing convincing evidence that DM can initiate or accelerate the development of atherosclerosis. The relationship is strong: elevated blood glucose levels, dyslipidemia, and accompanying metabolic changes play a significant and undeniable role in the pathogenesis of atherosclerosis at various stages of atherosclerotic plaque formation in DM patients [5,6,7].

To grasp the fundamental principles behind this phenomenon, a systems approach incorporating Petri nets was employed [8,9,10], as elaborated in this paper. Such an approach to a complex process, which atherosclerosis coexisting with DM certainly is, facilitates the organization of knowledge and increases the understanding of the biological system by building a mathematical model that represents a complex network of interactions [9].

This study aimed, based on the created model of interaction pathways between hyperglycemia and atherosclerosis, to detect molecules or processes in the studied system, the blocking of which could effectively inhibit or at least weaken atherosclerosis progression.

### 1.2. Biological Background

**Figure 1:**
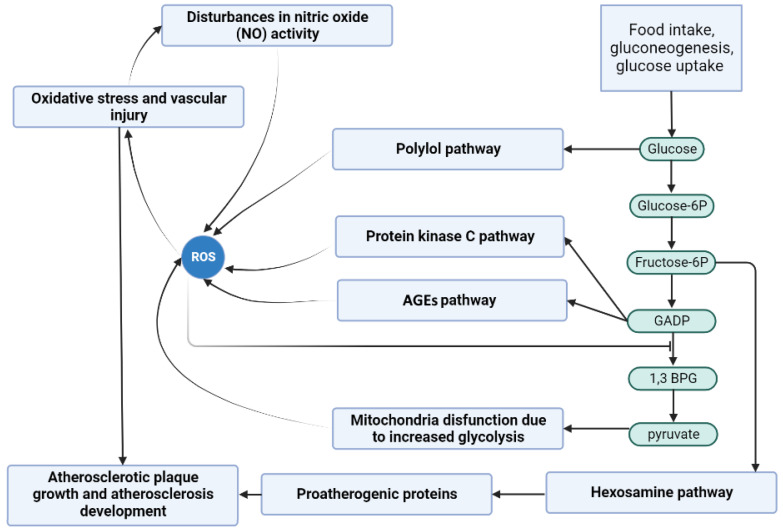
Diagram of the role of carbohydrate metabolism disorder in atherosclerosis development and progression.

The diagram below (see Figure 1) presents the most significant processes that have been taken into account in the Petri net-based model presented in this paper. It will be discussed in detail in this section.

#### 1.2.1. Glycolytic Pathway

The glycolytic pathway, known as glycolysis (see Figure 1), is a fundamental metabolic pathway that occurs in the cytoplasm of cells. It serves as a central energy-generating process as it involves the breakdown of glucose into two pyruvate molecules in a series of enzymatic reactions. The process begins with the phosphorylation of glucose, catalyzed by the enzyme hexokinase, resulting in the formation of glucose-6-phosphate (glucose-6P). Glucose-6P is then converted into fructose-6-phosphate (fructose-6P) through an isomerization reaction. Next, fructose-6P undergoes phosphorylation and cleaves into glyceraldehyde-3-phosphate (glyceraldehyde-3P) and dihydroxyacetone phosphate (DHAP). Glyceraldehyde-3P is further metabolized, which involves a series of reactions, including the conversion of glyceraldehyde-3P to 1,3-diphosphoglycerate (1,3-BPG), catalyzed by the enzyme glyceraldehyde-3P dehydrogenase (GAPDH). NAD${}^{+}$ (nicotinamide adenine dinucleotide) is reduced to NADH (reduced nicotinamide adenine dinucleotide) during this process. Following its formation, 1,3-diphosphoglycerate (1,3-BPG) sets off on a cascade of reactions orchestrated by various enzymes, culminating in pyruvate production [6,11]. Subsequently, pyruvate is transported to the mitochondria, entering the tricarboxylic acid (TCA) cycle and generating carbon dioxide (CO${}_{2}$), NADH, and flavin adenine dinucleotide (FADH${}_{2}$) molecules, thereby contributing to mitochondrial dysfunction [6,12,13].

Next, NADH and FADH${}_{2}$ donate electrons to the electron transport chain (ETC) in mitochondria for adenosine triphosphate (ATP) production. Electrons pass through complex III, cytochrome C, complex IV, and finally to oxygen, producing water. The voltage across the mitochondrial membrane drives ATP synthesis. High glucose levels enhance glycolysis, increasing electron donation to the ETC. This elevation in electron flux raises the membrane potential, leading to increased electron donation to oxygen, generating reactive oxygen species (ROS), specifically superoxide anion ($O_{2}^{•}-$) [12,13].

#### 1.2.2. Reactive Oxygen Species

ROS and reactive nitrogen species (RNS) are products of cellular metabolism. They are known for having a harmful or beneficial role depending on their concentration in tissues. ROS (see Figure 1) disrupt the function of endothelial nitric oxide synthase (eNOS), thus promoting the development of atherosclerosis and escalating the generation of other ROS, particularly the superoxide anion ($O_{2}^{•}-$) in endothelial cells and vascular smooth muscle cells [14]. The superoxide anion, in its initial stages, reacts with nitric oxide (NO) to generate peroxynitrite ($ONOO^{-}$), a potent oxidative agent that selectively inhibits prostacyclin (PGI${}_{2}$). As a consequence, the inactivation of PGI${}_{2}$ leads to the accumulation of its precursor, prostaglandin endoperoxide ($PGH_{2}$), triggering vasoconstriction and impairing endothelial function. Furthermore, $PGH_{2}$ fosters the conversion of PGI${}_{2}$ to thromboxane $A_{2}$ ($T_{x}A_{2}$) through the action of $T_{x}A_{2}$ synthase. Both of these occurrences activate the $T_{x}A_{2}$ receptor, instigating platelet aggregation, as well as inducing the activation of vascular smooth muscle cells, apoptosis, and the expression of pro-inflammatory adhesion molecules, including intercellular adhesion molecule-1 (ICAM-1), vascular cell adhesion molecule-1 (VCAM-1), and endothelial-leukocyte adhesion molecule (ELAM-1) [15,16,17].

Peroxynitrite also disrupts eNOS, decreasing NO synthesis and increasing ROS production. Furthermore, superoxide inactivates glyceraldehyde-3-phosphate dehydrogenase (GAPDH), thereby contributing to vascular injury via the protein kinase C (PKC), hexosamine, polyol, and advanced glycation end products (AGEs) pathways [6].

#### 1.2.3. Protein Kinase C (PKC) Pathway

The PKC pathway (in DM, the most engaged are PKC β and γ; see Figure 1) is a complex cascade of events involving the activation and regulation of protein kinase C enzymes. First, DHAP is converted to glycerol-3-phosphate through the action of the enzyme glycerol-3-phosphate dehydrogenase. Glycerol-3-phosphate is then acylated to form phosphatidic acid, which is subsequently dephosphorylated to generate diacylglycerol (DAG). DAG acts as a critical second messenger within the PKC pathway. It directly binds to and activates specific isoforms of PKC, particularly PKCβ and PKCγ, which are the isoforms most prominently associated with the development of diabetic atherosclerosis. Upon activation, PKC (β and γ) translocates to cellular membranes, where it phosphorylates target proteins, modulating their function and impacting various cellular processes [18,19].

The upregulation of PKC (β and γ) triggered by hyperglycemia significantly impacts endothelial cells, increasing their permeability, reducing nitric oxide production, and increasing synthesis of vasoconstrictors like endothelin 1 (ET-1) and thromboxane A2 (T${}_{x}$A${}_{2}$). Additionally, PKC (β and γ) contributes to the pro-inflammatory state observed in DM by enhancing the expression of nuclear factor-κB (NF-κB) in smooth muscle cells. Moreover, PKC (β and γ) disrupts the balance of ROS through the activation of membrane-associated NAD(P)H-dependent oxidases and the inhibition of insulin-stimulated eNOS production [6,19].

#### 1.2.4. Hexosamine Pathway

The hexosamine pathway (see Figure 1) branches off from glycolysis, and increased flux through this pathway has been observed in insulin-resistant states, contributing to altered insulin signaling and glucose uptake [20]. It begins with converting fructose-6-phosphate into glucosamine-6-phosphate, catalyzed by glutamine:fructose-6-phosphate aminotransferase (GFAT), a key enzyme in this pathway. Ultimately, the path leads to the production of UDP-N-acetylglucosamine (UDP-GlcNAc). UDP-GlcNAc, the end product of the hexosamine pathway, serves as a crucial substrate for adding N-acetylglucosamine residues to proteins and lipids through O-GlcNAcylation. The post-translational process of O-GlcNAcylation, followed by ubiquitination and degradation of essential atheroprotective proteins like eNOS, disrupts the delicate equilibrium, tilting it towards enhanced atherogenesis. Simultaneously, this process boosts the transcription of proatherogenic proteins, further exacerbating the development of atherosclerosis [6,20].

Additionally, hyperglycemia promotes ECs dysfunction, acting, at least in part, through glucosamine-induced endoplasmic reticulum (ER) stress. This results in increased apoptosis and increased expression of inflammatory and adhesion/prothrombotic molecules, which play a key role in vascular damage and contribute to atherosclerosis [21].

#### 1.2.5. Polyol Pathway

The polyol pathway (also known as the sorbitol or aldose reductase pathway) (see Figure 1) is a metabolic process occurring in the cytosol of various cells. In the first step of this pathway, glucose is reduced to sorbitol by the enzyme aldose reductase, utilizing NADPH as a cofactor. This conversion serves as a protective mechanism, helping to decrease glucose levels within cells during high glucose concentrations. The second step involves the oxidation of sorbitol to fructose, catalyzed by the enzyme sorbitol dehydrogenase (SDH), using NAD${}^{+}$ as a cofactor [22].

Excessive activation of the polyol pathway by persistent hyperglycemia can lead to the accumulation of sorbitol and fructose, which can have detrimental effects. It has been found that 30% of the body’s glucose is metabolized via this path, contributing to the NADH/NAD${}^{+}$ redox imbalance [23]. Increased production of ROS from the polyol pathway promotes DM complications through (1) hyperglycemia-induced loss of glutathione, (2) the accumulation of sorbitol, and (3) the increased osmotic stress as an effect of sorbitol accumulation. Moreover, NAD${}^{+}$ degradative pathways disrupt the NADH/NAD${}^{+}$ redox imbalance created in the polyol pathway [24].

In addition, fructose can promote the formation of AGEs, enhancing oxidative stress. Elevated levels of ROS strongly impact oxidative stress by depleting NADPH, a crucial cofactor essential for the regeneration of reduced glutathione (GSH) and a vital scavenger of ROS. It should be highlighted that the conversion of sorbitol to fructose generates an excess of NADH, thereby intensifying the overall oxidative burden [6,25].

#### 1.2.6. Advanced Glycation End Products (AGEs) Pathway

The AGEs pathway (see Figure 1) involves a series of reactions between glucose and proteins, resulting in the formation of AGEs, a complex group of compounds formed exogenously or endogenously by various mechanisms. AGEs are formed due to non-enzymatic condensation between the carbonyl groups of reducing sugars and free amino groups of nucleic acids, lipids, or proteins, followed by further rearrangements that strive to obtain stable and irreversible end-products. These AGEs accumulate in various tissues throughout the body, leading to cellular dysfunction, tissue damage, and accelerating vascular disease progression [26,27].

The AGEs pathway is influenced by the presence of methylglyoxal (MGO) and glyceraldehyde-3P, which are essential intermediates in the formation of AGEs. MGO is a reactive dicarbonyl compound formed primarily during glucose metabolism from glyceraldehyde-3P. Accumulation of glyceraldehyde-3P occurs secondary to the inhibition of glyceraldehyde-3P dehydrogenase (GAPDH). This accumulation contributes to the modification of glyceraldehyde-3P and the subsequent formation of AGEs [6,26].

The presence of MGO and glyceraldehyde-3P results in the modification of various intracellular and extracellular molecules. Extracellular AGEs can interact with transmembrane receptors known as RAGE (receptors for AGEs), expressed on endothelial cells, smooth muscle cells, macrophages, and lymphocytes. Activation of RAGE can trigger cellular responses such as increased generation of ROS and the induction of pro-inflammatory states through the upregulation of NF-κB and VCAM-1 [27]. The AGE-RAGE signaling pathway, a complex and intricate cascade, plays an essential role in the pathogenesis of diabetic complications (see [28]). In a PKC-dependent manner, RAGE recruits two adaptor proteins, differentiation factor 88 (MyD88) and the toll-interleukin-1 receptor (TIR) domain-containing adaptor protein (TIRAP), forming multiprotein complexes required for downstream action [28]. Thus, RAGE-binding ligands activate protein kinase B (AKT) via TIRAP/MyD88, perpetuating NF-κB activation, thereby enhancing the pro-inflammatory response. In addition, RAGE ligation increases the NADPH oxidase subunits’ expression and activates NADPH oxidase, contributing to the ROS generation.

#### 1.2.7. Oxidative Stress

DM vascular complications are associated with increased ROS production. The endothelium, a metabolically active single-layered organ, is located in a strategic place, constituting a barrier between the blood and all tissues. This organ is exposed to various stimuli, to which it responds, to maintain the integrity and homeostasis of vascular function [29]. Due to their inability to modulate intracellular glucose concentration relative to blood glucose concentration, endothelial cells are one of the main targets of hyperglycemic injury. During hyperglycemia, they cannot prevent glucose from entering; therefore, the endothelium contains high glucose levels and may be subject to significant oxidative stress. Damage caused by ROS and by AGEs can trigger an endothelial inflammatory response. Other mechanisms may also play an essential role, such as the harmful effect of AGEs on RAGE. Their binding signals the intracellular formation of ROS and the activation of transcription factors. As a result of oxidative stress developing in cells, the pro-inflammatory transcription factor NFκB is activated, as well as activation of various signaling pathways. Under these conditions, many cytokines and growth factors also increase. By activating various reductases, including AR, the occurring phenomena accelerate the formation of further glycation end products and intensify the stress. There is also an increase in the expression of adhesion molecules. Oxidative stress, as an imbalance between the generation and removal of ROS, harmfully affects insulin activity [30]. It has been proposed as the etiological factor responsible for insulin resistance, beta cell dysfunction, and impaired glucose tolerance, ultimately leading to type 2 diabetes mellitus (T2DM) [24]. Furthermore, it has been found that there is increased oxidative stress in response to postprandial hyperglycemia in patients with T2DM [31]. Animal studies have revealed that the upregulation of Nox1, the predominant isoform in the vascular cells of the NADPH oxidase, contributes to the development of diabetic atherosclerosis; hence, reducing its expression can alleviate disease progression [32,33]. Moreover, experimental evidence has confirmed the involvement of oxidative stress in diabetes-related atherosclerosis. Mice deficient in Gpx1, a key regulator of antioxidant enzymes, exhibit an accelerated formation of plaques, which is mitigated upon restoration of Gpx1 levels [34]. These findings highlight the potential therapeutic significance of targeting ROS and oxidative stress in managing diabetes-associated atherosclerosis [32,33].

#### 1.2.8. Diabetes Mellitus and Atherosclerosis

So far, it has not been possible to demonstrate a direct pro-atherogenic effect of hyperglycemia on cells present in lesions of atherosclerosis in vivo [35]. It remains likely that elevated glucose acts primarily on tissues, including the liver or adipose tissue, and the effect on cells in atherosclerotic lesions is due to changed signaling from these tissues. Elevated intracellular glucose levels increase flow through cellular metabolic pathways, such as the mitochondrial electron transport system, resulting in ROS overproduction. In addition, glucose metabolites can induce pro-inflammatory reactions by activating protein kinase C-β and AR. Hence, there is agreement that diabetes and atherosclerosis are interconnected through several pathological pathways. Studies involving patients with diabetes have shown an increased risk and accelerated development of atherosclerosis [36]. For instance, several studies have reported the early development of atherosclerosis in adolescents and children with type 1 diabetes mellitus (T1DM) [37]. Dyslipidemia with increased levels of atherogenic low-density lipoproteins (LDL) and elevated hepatic production of triglyceride-rich lipoproteins accompanied by hyperglycemia, endothelial dysfunction, oxidative stress, and increased inflammation have been proposed as factors explaining this acceleration (see [38]). However, diabetes-related atherosclerosis (see Figure 1) follows a histologic course comparable to atherosclerosis in individuals without DM. The progression encompasses multiple stages, including endothelial injury, a proliferation of smooth muscle cells, formation and infiltration of foam cells, activation of platelets, and heightened inflammation [38].

Local endothelial dysfunction is considered the instigating factor in the cascade of pathological events leading to the development of atherosclerosis [5]. It causes heightened permeability, retaining harmful low-density lipoproteins (LDL) that interact with the underlying extracellular matrix (ECM). This interaction sequesters LDL particles within the vessel wall, facilitating its oxidation by ROS. The oxidized LDL stimulates the overlying endothelial cells to upregulate cellular adhesion molecules, chemotactic proteins, and growth factors and inhibits NO production. These responses recruit monocytes and macrophages, interacting with highly oxidized aggregated LDL to form foam cells. Activated macrophages further contribute to this process by producing pro-inflammatory cytokines and stimulating the proliferation of vascular smooth muscle cells [7,36]. Atherosclerotic plaques narrow the arterial lumen, leading to ischemia and metabolic alterations in the affected tissues. The potential for thrombus formation triggered by unstable plaques is of even more significant concern. These thrombotic events can carry grave consequences, including fatal outcomes [38].

## 2. Materials and Methods

Petri nets are well suited for modeling and analysis of complex biological systems. They are mathematical objects similar to graphs. To be more precise, they have a structure of directed weighted bipartite graphs. In other words, these nets are composed of vertices of two types, called places and transitions. An arc can connect only a place with a transition or vice versa. When a Petri net is a model of a biological system, transitions are counterparts of active components of the system; they represent some elementary processes occurring in the system (e.g., chemical reaction), while places are counterparts of passive components (e.g., chemical compounds). Arcs describe causal relationships between the active and passive components [9,39,40].

The already described elements of Petri nets constitute their structure, but there is one more type of the nets’ components that brings to them a kind of dynamics. These components are tokens located in places. They can flow from one place to another via transitions, and the flow of them represents a flow of signals, substances, information, and so on, through the modeled system. Tokens residing in places represent amounts of passive components modeled by these places.

From the existence of tokens, there are many important properties of Petri nets. They cause nets of this type to be very useful for modeling the behavior of concurrent systems (which would be hard or even impossible using graphs).

The flow of tokens follows the transition firing rule, according to which a transition is active if, in every place directly preceding it, the number of residing tokens is equal to or greater than the weight of an arc connecting a given place with the transition. An active transition can be fired, which means that tokens can flow from the places directly preceding it to the ones directly succeeding the transition. The numbers of flowing tokens equal the weights of respective arcs [39,40].

Petri nets have an intuitive graphical representation, being very useful at the stages of model development and simulation. In this representation, places are depicted as circles, transitions as rectangles, arcs as arrows, and tokens as dots or numbers located in places [9].

There are a lot of mathematical methods and software tools for a formal analysis of Petri nets’ properties. However, the graphical representation is not well suited for such analysis. For this purpose, another one is used, that is, an incidence matrix. In such a matrix *A*, rows correspond to places, columns to transitions, and entry $a_{ij}$ is equal to a change in the numbers of tokens residing in place $p_{i}$ when transition $t_{j}$ is fired [9,41].

The analysis of Petri net-based models of biological systems can be based on t-invariants, which are vectors $x\in Z^{m}$, where *m* is the number of transitions, satisfying equation A·x=0. With every t-invariant *x*, there is an associated set of transitions $s\left(x\right)=\left\{t_{j}:x_{j}>0,j=1,2,\ldots ,m\right\}$, called its support. If every transition $t_{j}\in s\left(x\right)$ is fired $x_{j}$ times, the distribution of tokens over the set of transitions does not change. Since this distribution (called marking) corresponds to a state of the modeled system, t-invariants correspond to subprocesses occurring in the system, which do not change its state. A Petri net, being a model of a biological system, should usually be covered by t-invariants, meaning that every transition should belong to at least one t-invariant support [9,41].

Transitions can be grouped into disjoint sets called MCT sets (maximum common transition sets). Such a set contains those transitions that are elements of exactly the same t-invariant supports. Non-trivial MCT sets (i.e., those that contain more than one transition) correspond to some functional blocks of the modeled system [41,42].

An analysis of Petri net-based models of biological systems can also be based on disabling selected parts of the model and observing the behavior of other parts [43,44]. Such an analysis is called knockout analysis, and two variants of it can be distinguished. In one of them, which is based on an analysis of t-invariants, selected transitions are disabled, and as a result, some t-invariants disappear. The goal of this type of analysis is to identify subprocesses affected by knocking out selected elementary processes (transitions). The second variant is based on simulation. In this case, chosen transitions are disabled, and the resulting distribution of tokens over the set of places and the firing rates of remaining transitions are analyzed. To do this, a series of simulations starting with the same initial conditions is performed. The goal is to examine the influence of some elementary processes (represented by single transitions) or groups of them on the behavior of the analyzed system.

**Figure 2:**
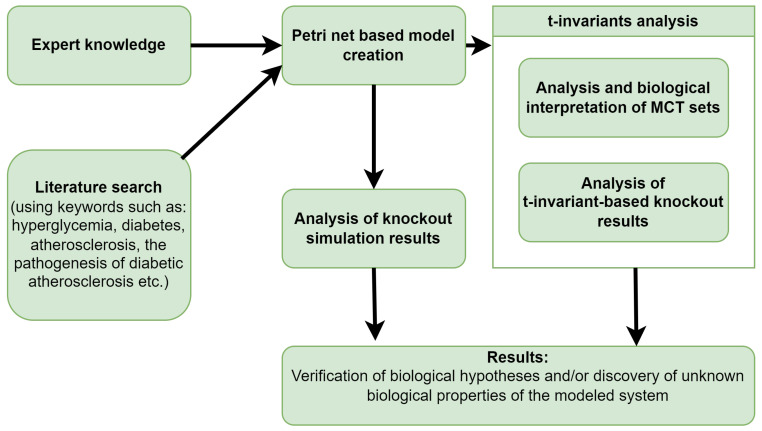
Detailed operational framework setting forth the essential procedures for building a model using Petri nets, analyzing the model, and achieving the intended results.

Figure 2 provides a straightforward diagram that encapsulates all the stages, from the inception of the model to the derivation of the results.

All analyses, including the calculation of t-invariants, MCT sets, t-invariant-based knockout analysis, and image preparation, were performed using Holmes software, version 1.1 [45]. The exception was the simulation knockout, aimed at estimating the importance of each functional biological activity (represented by both non-trivial and trivial MCT sets) of the net, which was executed with MonaLisa [46]. Nevertheless, the results from this simulation were subsequently analyzed in Holmes. For in-depth technical insights on the analysis process and image creation, an interested reader can refer to the Holmes manual [47].

## 3. Results and Discussion

In this section, a Petri net-based model of the interaction pathways between DM and atherosclerosis, along with its analysis, is presented. It is worth noting that the model’s foundation is built upon data exclusively derived from the established and peer-reviewed biological and medical literature. This implies that the initial design and structure of the model are based on previously published experimental and clinical findings.

The model was analyzed using t-invariants, MCT sets, and through knockout analysis. By employing established Petri net methodologies, a solid theoretical foundation for the conclusions is ensured. The systematic nature of our modeling and analysis methods allows for replication by other researchers using identical data sources and tools. While the conclusions are rooted solely in silico analyses, they are consistent with experimental and clinical literature.

### 3.1. Petri Net-Based Model Presentation and the Results of Its Formal Analysis

**Figure 3:**
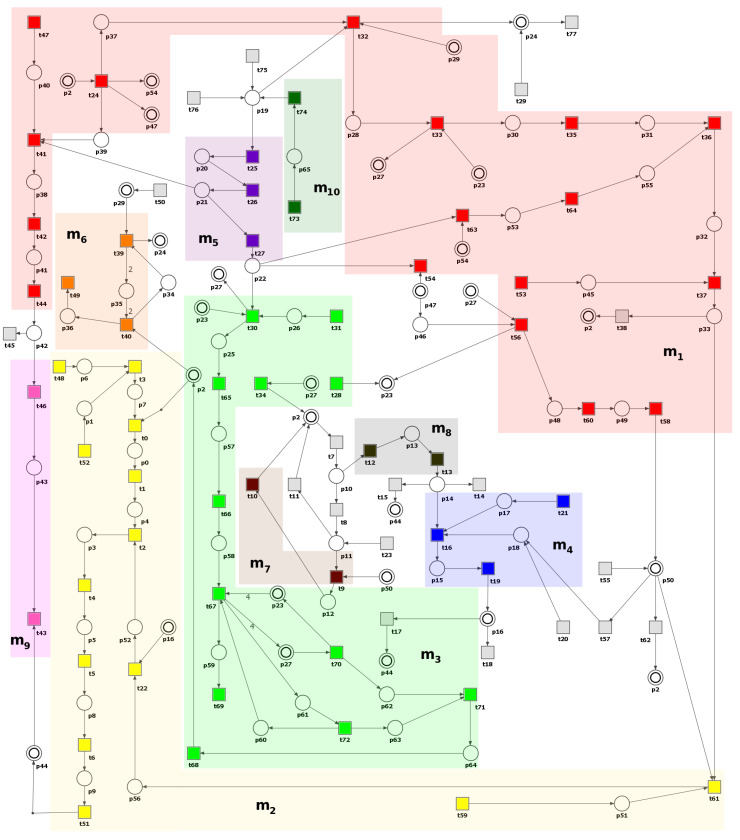
Petri net-based model of the role of carbohydrate metabolism disorder in atherosclerosis development and progression divided into 10 subprocesses according to MCT sets, marked with different transition colors (see Table 1 for more detailed descriptions of these subprocesses). Transitions are represented by squares, and places by circles. The places that are presented as two concentric circles correspond to the same place in the model and are called logical places. The figure was created using Holmes [45].

The Petri net-based model of the role of carbohydrate metabolism disorder in atherosclerosis development and progression has been created using a tool called Holmes [45]. It is presented in Figure 3 and consists of 66 places (biological components) and 78 transitions (elementary processes), whose names are listed in Table A1 and Table A2, respectively.

Furthermore, there are 150 minimal t-invariants covering all transitions in the net (i.e., each transition belongs to the support of at least one t-invariant). Based on t-invariants, MCT sets have been calculated. The model contains 10 non-trivial MCT sets (i.e., those having more than one transition). Table 1 shows these sets and their biological meaning. The results were obtained using Holmes [45].

### 3.2. Knockout Analysis Based on t-Invariants

**Scenario 1.**  *Analysis of the importance of each functional biological unit (MCT set) and some selected transitions in the considered model*.

As a next step, we have conducted the knockout analysis of the studied model. First, to estimate the importance of each functional biological activity (represented by non-trivial and trivial MCT sets) of the net, each of them has been knocked out. The impact of turning off a single unit on the model was measured by the number of transitions affected by such a knockout. A transition is affected by a knockout if it is present only in the supports of the affected t-invariants (i.e., t-invariants that have knocked-out transitions in their supports). For example, if transition $t_{x}$ is knocked out, then transition $t_{y}$ is not considered to be affected by $t_{x}$ if it is present in at least one t-invariant that does not contain $t_{x}$ in its support.

The results of the knockout analysis are given in Table 2 (single transitions that have a significant effect, i.e., affecting more than 2% of all transitions, have been shown). Since knocking out any transition belonging to an MCT set has exactly the same impact on t-invariants, two values are given for each MCT set in Table 2. The first one provides the percentage of all disabled transitions, including those comprising the MCT set, while the second one, given in parenthesis, is the percentage of transitions outside of the knocked-out MCT set that are also disabled.

The results indicate that the most critical to the analyzed model are the following pathways: protein kinase C pathway, NADP usage, NADPH formation, ROS reaction with NO, and atherosclerosis development affected by ROS and oxidized LDL. All these processes contribute to the onset and progression of atherosclerosis and the associated oxidative stress.

Furthermore, recent research has revealed that hyperglycemia induces oxidative stress by promoting the production of ROS. This phenomenon is initiated by the activation of diverse molecular pathways, including PKC (β and γ) activation, advanced glycation end products (AGEs) formation, enhanced polyol pathway flux, and hyperactivation of the hexosamine pathway [1,48]. Thus, the results obtained are in line with those of recent studies.

**Table 2:** The impact of a knockout of selected net elements (MCT sets or single transitions) depending on the percentage of affected transitions, calculated on the basis of both approaches described in [49] and simulation knockout (identically as in [43,44]).

MCT Set/Transition	Biological Function	Affected Transitions
$m_{1}$	Protein kinase C pathway	69.23% (48.75%)
$t_{77}$	NADP usage	52.56%
$t_{50}$	NADPH formation	50.00%
$m_{2}$	Atherosclerosis development and progression affected by ROS and oxidized LDL	44.87% (29.51%)
$t_{7}$	ROS reaction with NO	34.62%
$m_{6}$	Antioxidant defense mechanism involving glutathione-dependent enzymes	32.05% (29.49%)
$m_{4}$	Activation of the thromboxane receptor leading to apoptosis, vascular muscle cell activation, and vascular cell adhesion molecules expression	24.36% (21.80%)
$m_{9}$	Activation of proatherogenic proteins leading to atherosclerosis progression	20.51% (19.23)
$m_{7}$	Impact of peroxynitrite on nitric oxide (NO) synthesis	15.38% (14.10%)
$m_{8}$	Vasoconstriction and endothelial dysfunction induced by PGI${}_{2}$ synthase disruption and inactivation	14.10% (12.82%)
$t_{38}$	ROS production by AGE-RAGE complex	3.85%
$m_{5}$	Metabolic pathway that converts glucose into glyceraldehyde 3-phosphate	2.56% (0%)
$m_{3}$	Increased production of ROS as a consequence of mitochondrial dysfunction resulting from hyperglycemia	1.28% (0%)
$m_{10}$	Glucose uptake and transport across the cell membrane	1.28% (0%)

**Scenario 2.**  *Inhibition of PKC (β and γ) pathway in glucose metabolism*.

Many studies have established that hyperglycemia-induced atherosclerosis is significantly influenced by the activation of PKC (β and γ), which induces various cellular responses, including the expression of growth factors, activation of different signaling pathways, and increase in oxidative stress [19]. In addition, impaired antioxidant defense can cause persistent high levels of ROS, leading to inflammatory and apoptotic responses in diabetic cells. PKC (β and γ)-dependent activation of nicotinamide adenine dinucleotide phosphate (NADPH) oxidase contributes to increased ROS generation. It has been shown that inhibiting PKC (β and γ) can reduce the induction of NADPH oxidase in a high-glucose environment [48].

**Figure 4:**
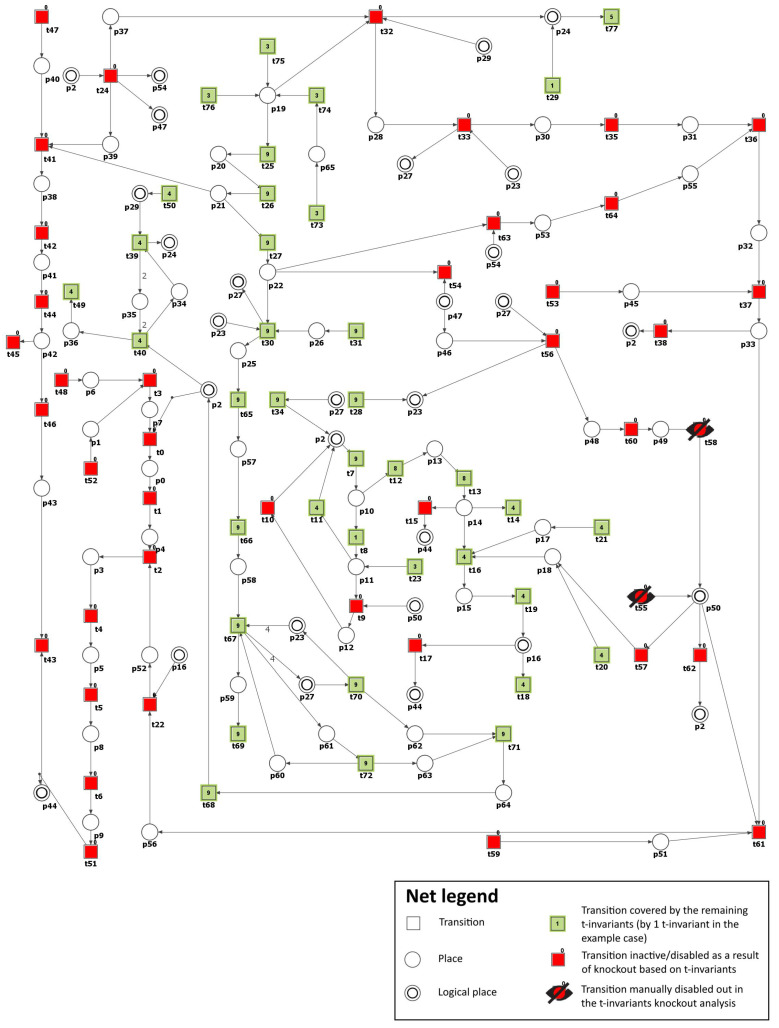
Illustration of the impact of PKC (β and γ) inhibition. It is shown as a graphical representation of the t-invariant-based knockout impact of the transitions $t_{55}$ (PKC (β and γ) expression) and $t_{58}$ (PKC (β and γ) activation by DAG) on atherosclerosis progression ($t_{43}$). The knocked-out transitions are depicted as a crossed-out black circle. Transitions that belong to the support of any t-invariant are denoted as filled-in green rectangles. Other transitions (not belonging to the support of any t-invariant) are represented as filled-in red rectangles. The numbers shown inside the green rectangles correspond to the number of supports of t-invariants to which a given transition belongs. The results and the figure were obtained using Holmes [45].

To simulate the impact of PKC (β and γ) inhibition, we have removed the following transitions from the net: $t_{55}$ (PKC (β and γ) expression) and $t_{58}$ (PKC (β and γ) activation by DAG). Consequently, the number of t-invariants that contribute to the progression of atherosclerosis decreased from 90 to 0 (see Figure 4 and Table 3), as observed. This implies that the development of atherosclerosis has been halted in the modeled system.

In addition, by knocking out transitions $t_{55}$ and $t_{58}$, the following transitions and MCT sets were excluded: $m_{1}$, $m_{2}$, $m_{7}$, $m_{9}$, $t_{15}$, $t_{17}$, $t_{38}$, $t_{45}$, $t_{55}$, $t_{57}$, $t_{62}$, which are engaged in the activation of proatherogenic proteins, NADPH oxidase by protein kinase C, atherosclerosis progression affected by ROS, and degradation of atheroprotective proteins. Simultaneously, the glucose metabolism and the defense mechanism against oxidation, which includes enzymes dependent on glutathione, remained unaffected. The oxidative stress was significantly decreased but was still observed, with 13 out of 149 t-invariants remaining, as calculated by Holmes [45] (based on place $p_{2}$ (ROS)).

These findings align with the results presented in [19]. However, although researchers have revealed the potential effect of PKC inhibition in attenuating the progression of microvascular complications in diabetes, only the PKC β inhibitor ruboxistaurin (RBX) has shown potential for use in diabetic retinopathy. Still, other PKC inhibitors have not shown satisfactory results [50]. RBX has undergone phase III clinical trials and is awaiting approval for treating diabetic retinopathy in the U.S. and Europe [51]. While more profound studies are needed to confirm the findings, the ongoing studies suggest that PKC (β and γ) may hold promise not only in the context of diabetic retinopathy but also, in general, for atherosclerosis associated with diabetes.

**Scenario 3.**  *Inhibition of polyol pathway in glucose metabolism*.

In health conditions, when glucose homeostasis is maintained, the polyol pathway constitutes a secondary route of glucose metabolism that operates in parallel with glycolysis. However, glucose flux through this pathway increases significantly during hyperglycemia, resulting in excessive sorbitol formation and accounting for up to 30% of total glucose consumption. This leads to the depletion of reducing equivalents and accumulation of osmotically active polyols [52].

Aldose reductase (AR) is the first enzyme of the polyol pathway catalyzing the conversion of glucose to sorbitol. In hyperglycemia, AR reduces high levels of intracellular glucose to sorbitol, and this reaction consumes NADPH, a cofactor necessary to regenerate reduced glutathione, a key antioxidant. Therefore, decreasing the level of reduced glutathione, AR increases the intracellular potential for oxidative stress. In addition, DM is associated with platelet hyperactivity, and AR is involved in it since it was found to synergistically modulate the platelet response to both hyperglycemia and collagen exposure in human platelets through a signaling pathway involving ROS, PKC, and MAPK [53]. Human AR expression in transgenic mice has been observed to accelerate DM-related atherosclerosis, suggesting that AR may play an important role in atherothrombosis [54]. Moreover, AR gene polymorphisms have been found to be associated with most diabetic complications [55].

Apart from being involved in diabetic complications, AR also plays several other physiological roles, which include (1) the production of intermediates facilitating the production of AGE precursors; (2) a process that diverts glucose from glycolysis and glucose oxidation; (3) participation in the metabolism of steroids, norepinephrine intermediates, aldehyde detoxification or glutathionylated derivatives; and (4) modulating lipid metabolism through the ability of AR to regulate histone deacetylase 3 (HDAC3) degradation and consequently PPARγ activation [55]. However, there are still conflicting reports regarding AR’s roles in mammals in physiological and pathophysiological conditions. On the one hand, previous studies have suggested a beneficial role for AR in the detoxification of toxic lipid aldehydes produced by oxidative stress; on the other hand, the accelerated flux of sorbitol through the polyol pathway, thanks to AR, and the resulting increase in oxidative stress observed in DM is strongly associated with the development of secondary complications of DM. The fact that AR, aldehyde reductases, and aldehyde dehydrogenases can generally compete for different aldehydes makes it challenging to determine the specific physiological role of AR.

Due to numerous properties, AR has recently gained attention, and its inhibitors are of pharmacotherapeutic interest to the pharmaceutical community as potential and promising agents down-regulating major inflammatory pathologies [25,56,57]. AR inhibitors such as epalrestat have been shown to protect against diabetic peripheral neuropathy by mitigating oxidative stress and inhibiting polyol pathways [58].

**Figure 5:**
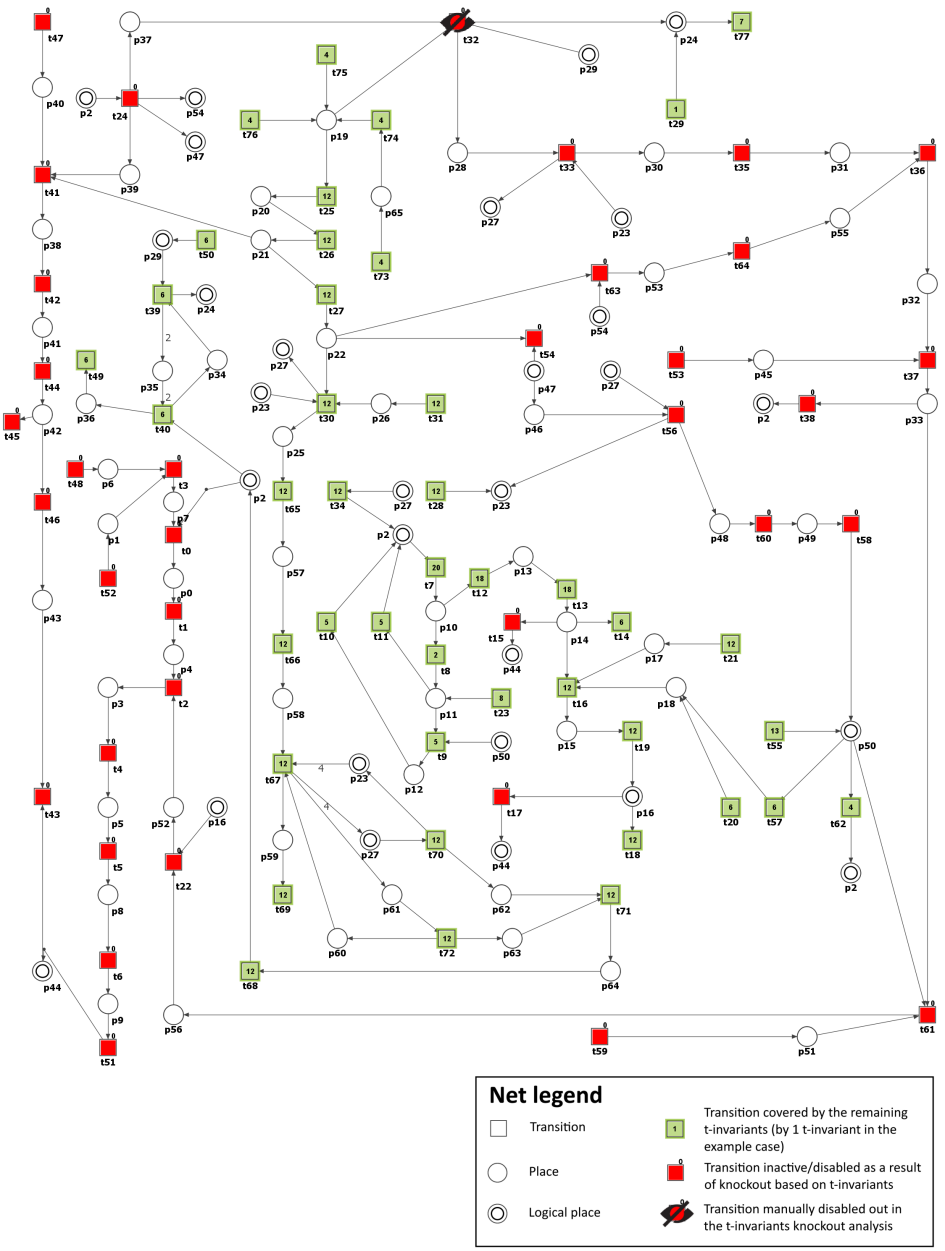
Illustration of the impact of polyol pathway inhibition. It is shown as a graphical representation of the t-invariant-based knockout impact of the transition $t_{32}$ (reaction catalyzed by aldose reductase) on atherosclerosis progression ($t_{43}$). The knocked-out transition is depicted as a crossed-out black circle. Transitions that belong to the support of any t-invariant are denoted as filled-in green rectangles. Other transitions (not belonging to the support of any t-invariant) are represented as filled-in red rectangles. The numbers shown inside the green rectangles correspond to the number of supports of t-invariants to which a given transition belongs. The results and the figure were obtained using Holmes [45].

To model the effect of AR inhibitors, the formation of sorbitol ($p_{28}$) has been blocked by excluding from the net transition $t_{32}$ (reaction catalyzed by aldose reductase). As a result, it was possible to observe that the number of t-invariants contributing to the atherosclerosis progression dropped from 90 to 0 (see Figure 5 and Table 3). This means that atherosclerosis development has been stopped.

Moreover, the knockout of transition $t_{32}$ has prompted the exclusion of the following transitions and MCT sets: $m_{1}$, $m_{2}$, $m_{9}$, $t_{15}$, $t_{17}$, $t_{38}$, $t_{45}$, which are involved in the activation of pro-atherogenic proteins, PKC (β and γ) pathway, and atherosclerosis progression affected by ROS and oxidized LDL. Similarly, as in Scenario 2, glucose metabolism and the antioxidant defense mechanism involving glutathione-dependent enzymes stayed intact. AR has a low affinity for glucose. Therefore, in hyperglycemia, there is an increased flux of glucose metabolism via the polyol pathway, enhancing oxidative stress. In our model, the oxidative stress decreased but remained present (26 out of 149 remaining t-invariants, calculated using Holmes [45], based on place $p_{2}$ (ROS)).

These findings are consistent with the results presented in [59,60], where it was shown that AR blockage could prevent atherosclerosis. Additionally, it was presented in [61] that AR inhibition led to decreased expression of PKC (β and γ) enzyme in the course of diabetes. Therefore, AR inhibitors (ARIs) represent a promising therapeutic approach for treating a wide range of diabetic complications.

Based on the premise that AR inhibition can prevent numerous diabetic complications, a number of AR inhibitors have been developed and tested; however, their clinical usefulness was found to be low. In addition, a recent study has shown that AR detoxifies aldehydes formed by the lipids’ peroxidation and their glutathione conjugates. Although, in some situations, this antioxidant function helps AR protect against tissue damage and dysfunction, the metabolic transformation of glutathione conjugates with aldehydes derived from lipid peroxidation can also produce reactive metabolites, stimulating mitogenic and inflammatory processes. Thus, AR inhibition may have both beneficial and harmful effects. However, accumulating evidence suggests that AR inhibition may modify the effects of cardiovascular disease, asthma, sepsis, and cancer; therefore, additional studies are needed to target AR inhibitors to specific disease states [25,57].

Mechanisms examining cellular and in vivo studies to establish the link between AR and inflammation have revealed that flux through impaired AR drives changes in inflammatory genes via early growth response protein-1 (Egr-1). AR activity and flux changes reduce NAD${}^{+}$ levels, decreasing NAD${}^{+}$-dependent deacetylase Sirtuin 1 (Sirt-1) activity and subsequent acetylation and Egr-1 expression in hyperglycemic states. These data established the existence of a new AR-SIRT1-EGR1 mechanism by which glucose may lead to a pro-inflammatory and pro-thrombotic response in hyperglycemia-induced atherosclerosis [55]

**Scenario 4.**  *Inhibition of the advanced glycation end-products (AGEs)*.

AGEs are a chemically diverse group of compounds, formed non-enzymatically as a result of condensation between the carbonyl groups of reducing sugars and free amino groups of proteins, lipids, and nucleic acids [62]. They are created exogenously or endogenously via various pathways, specifically, the Maillard reaction, polyol pathway, and oxidation reactions. A moderate presence of AGEs has been observed among healthy individuals, whereas in hyperglycemia, due to the increased glucose availability, their formation increases. According to one hypothesis based on the “glycometabolic theory”, early hyperglycemia leads to a proportional increase in the formation of AGEs and oxidative stress. Over time, proteins of the mitochondrial respiratory chain become increasingly glycated, leading to mitochondrial DNA damage and promoting a self-perpetuating cycle of AGE formation and oxidative stress independent of hyperglycemia [63]. At the cellular level, AGEs, through interaction with their receptors, mostly RAGE (found on the various cells’ surface, like monocytes and macrophages, endothelial cells, smooth muscle cells, and fibroblasts) modulate properties of the cells by (1) activation of signaling cascades (including extracellular signal-regulated kinase 1/2 (ERK 1/2), MAPK, NF-κB, Janus kinases (JAKs), and signal transducer and activator of transcription proteins (STATs) (STATs)), leading to ROS generation and oxidative stress development; (2) contribution to the inflammatory process via generating many proinflammatory cytokines and vascular adhesion molecules; and (3) promotion of autophagy and apoptosis. In addition, AGEs cause covalent modifications and cross-linking of serum proteins and ECM, changing their structure, stability, and functions, which disrupts cell–matrix and matrix–matrix interactions, influencing intercellular signaling and leading to profibrotic effects, reduced elasticity, and increased vascular stiffness, among other effects (see [64]). All this contributes to the acceleration of atherosclerosis in DM [62].

Recent population-based research has demonstrated that AGE–protein cross-links are related to the pathogenesis of atherosclerosis and CVD [65]. Moreover, one study indicated that the accumulation of AGEs was independently linked with heart failure, with the association being more significant in individuals with diabetes [66].

The adverse impact of AGEs has become the basis for an intensive search for pharmacological agents that can interfere with glycation reactions and their consequences. Various compounds with diverse chemical structures have been identified as inhibitors of the AGEs’ formation and accumulation or disrupt the associated signaling pathways. Some of these agents have shown promising results in protecting against end-organ damage in chronic conditions such as diabetes [62,67]. Different drugs, like metformin or pioglitazone, have already shown the ability to block AGEs’ formation in in vitro studies [68]. ACE inhibitors used in hypertension were also capable of reducing AGEs’ formation, and an angiotensin II receptor blocker, telmisartan, downregulated RAGE mRNA levels and inhibited superoxide generation [69]. A number of other compounds also have AGE-lowering properties. The flavonoid quercitin, for instance, can trap glyoxal and MGO and thereby inhibit the AGEs’ formation [70]. Rosiglitazone, a peroxisome proliferator-activated receptor γ (PPARγ) agonist, also decreased AGE levels and increased serum levels of sRAGE [71], known for their properties to neutralize AGEs’ negative effects [72]). However, because the listed compounds have a broad spectrum of activity, it is not yet elucidated to what extent their AGE-reducing properties contribute to their beneficial effects.

To simulate the impact of AGE inhibitors on atherosclerosis in our model, transition $t_{63}$ (reaction forming MGO) was excluded from the network, which prevented the formation of MGO ($p_{28}$), a major precursor of AGE. Consequently, the number of t-invariants contributing to the progression of atherosclerosis decreased from 90 to 0, as shown in Table 3. This indicates that the progression of atherosclerosis has been significantly reduced. It should be underlined here that we are far from believing that blocking MGO will completely inhibit the formation of AGEs in the human organism. Moreover, we know that certain processes and effects of chemical compounds occurring in the human body cannot be completely eliminated by blocking their endogenous synthesis in vivo. In this case, it is known that MGO is not only an endogenous metabolite but can be delivered exogenously through food, drinks, air pollution, and cigarette smoke; hence, its complete blockage seems impossible. In addition, as known, there are many precursors of AGE [73]. Hence, in the organism, a single MGO blockade will never stop AGE formation. Our motivation to inhibit AGE formation by blocking its main source in the system was to show how AGE is crucial for atherosclerosis plaque progression in DM.

The obtained results are identical to those for Scenario 3 (see Figure 5 and Table 3). This suggests that targeting the AGE-RAGE axis could be a promising therapeutic approach for managing atherosclerosis in individuals with diabetes [66].

**Scenario 5.**  *Inhibition of NADPH oxidase*.

NADPH oxidase is a crucial enzyme responsible for the formation of ROS in human blood vessels. Typically, the expression of NADPH oxidases in non-phagocytic cells is relatively low. However, it can be significantly upregulated in response to stimuli such as hyperglycemia and hyperlipidemia, increasing ROS production and oxidative stress. Experimental and clinical studies suggest that NADPH oxidase plays a critical role in the initiation and progression of atherosclerosis [33].

**Figure 6:**
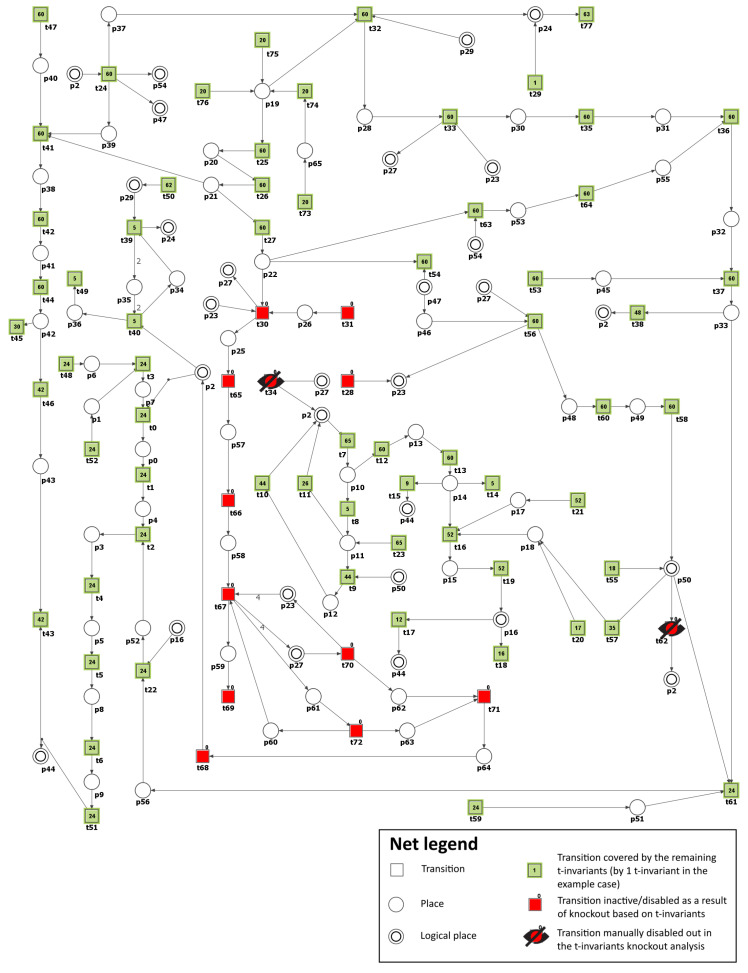
Illustration of the impact of NADPH oxidase inhibition. It is shown as a graphical representation of the t-invariant-based knockout impact of the transitions $t_{34}$ (ROS production via NADH oxidase by increased NADH) and $t_{62}$ (ROS production by activated NADPH-dependent oxidase) on atherosclerosis progression ($t_{43}$). The knocked-out transition is depicted as a crossed-out black circle. Transitions that belong to the support of any t-invariant are denoted as filled-in green rectangles. Other transitions (not belonging to the support of any t-invariant) are represented as filled-in red rectangles. The numbers shown inside the green rectangles correspond to the number of supports of t-invariants to which a given transition belongs. The results and the figure were obtained using Holmes [45].

To model the effects of inhibitors targeting NADPH oxidase, the transitions $t_{34}$ (ROS production via NADH oxidase by increased NADH) and $t_{62}$ (ROS production by activated NADPH-dependent oxidase) were removed from the network. As a result, the number of t-invariants associated with the advancement of atherosclerosis dropped from 90 to 42, as demonstrated in Table 3. This suggests a moderate slowdown in the development of atherosclerosis rather than a complete halt (see Figure 6). It is essential to highlight that, contrary to the scenarios mentioned above, the increased production of mitochondrial ROS associated with mitochondrial dysfunction has effectively ceased (see Figure 6). However, the analysis of the remaining t-invariants showed that the formation of AGEs still occurs, leading to the progression of atherosclerosis.

Building on this, when transitions $t_{34}$ and $t_{62}$, and also transition $t_{36}$ (increase in AGE formation), are knocked out, the number of t-invariants linked to the progression of atherosclerosis dramatically decreases from 90 to 0 (see Table 3). This suggests that, in this context, the progression of atherosclerosis is inhibited.

Furthermore, to evaluate the influence of inhibiting LDL oxidation in the studied model, in addition to transitions $t_{34}$ and $t_{62}$, the $t_{0}$ (oxidation) transition was deactivated. This transition is part of $m_{2}$, and the analysis performed in Scenario 1 revealed its considerable significance in the model. Consequently, the number of t-invariants associated with the advancement of atherosclerosis continued to decrease to 18, as illustrated in Table 3. This means that the progression of the disease has been further slowed down.

**Figure 7:**
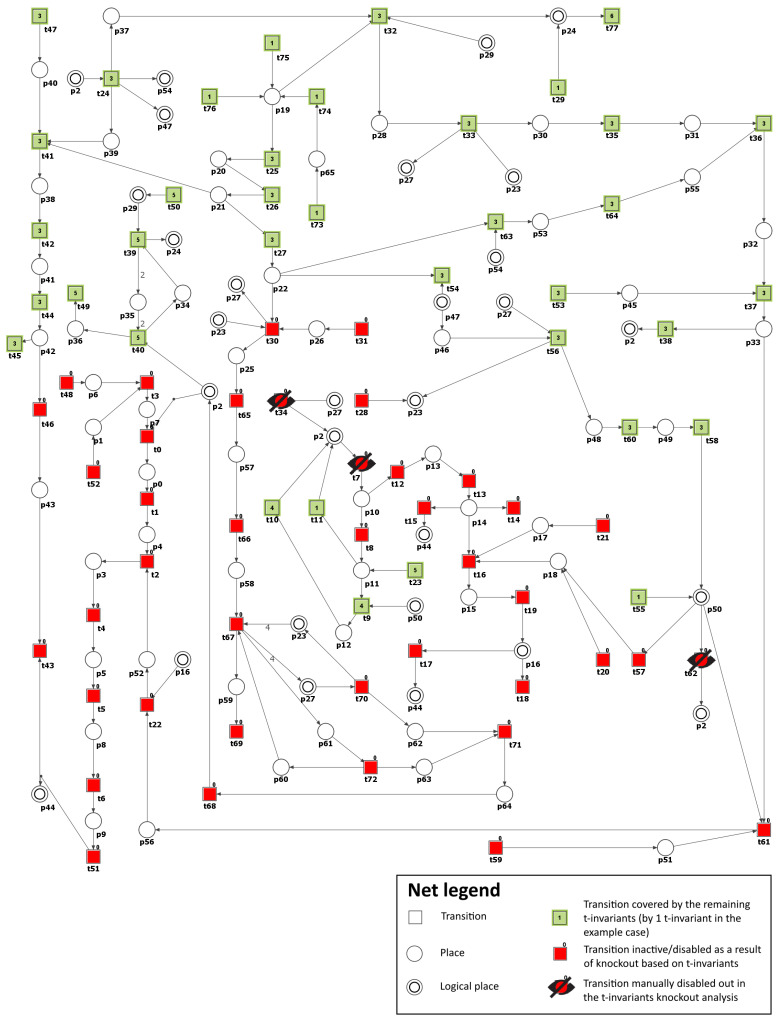
Illustration of the impact of NADPH oxidase and peroxynitrite formation inhibition. It is shown as a graphical representation of the t-invariant-based knockout impact of the transitions $t_{7}$ (ROS reaction with NO), $t_{34}$ (ROS production via NADH oxidase by increased NADH), and $t_{62}$ (ROS production by activated NADPH dependent oxidase) on atherosclerosis progression (t_43). The knocked-out transition is depicted as a crossed-out black circle. Transitions that belong to the support of any t-invariant are denoted as filled-in green rectangles. Other transitions (not belonging to the support of any t-invariant) are represented as filled-in red rectangles. The numbers shown inside the green rectangles correspond to the number of supports of t-invariants to which a given transition belongs. The results and the figure were obtained using Holmes [45].

According to Table 3, which presents the results of the analysis conducted within Scenario 1, another transition of significant importance and impact on the modeled phenomenon is $t_{7}$ (ROS reaction with NO). For this reason, as well, the $t_{7}$ transition was additionally deactivated, except for transitions $t_{34}$ and $t_{62}$. As a consequence, the number of t-invariants linked to the progression of atherosclerosis decreased significantly from 90 to 0 (see Table 3), indicating that the advancement of the disease has been completely halted (see Figure 7).

Furthermore, similar to Scenario 2, the integrity of glucose metabolism and the antioxidant defense mechanism, including glutathione-dependent enzymes, remained intact. Additionally, it is essential to highlight that, simultaneously, the oxidative stress has experienced a substantial decrease (5 out of 149 remaining t-invariants, calculated using Holmes [45] based on the place $p_{2}$ (ROS)).

In conclusion, the blockade of NADPH oxidase is a promising but challenging strategy. A more comprehensive understanding of NADPH oxidase’s roles in ROS formation and other cellular functions, along with the underlying mechanisms of atherosclerosis, might be necessary to make this approach more effective. Future research may need to focus on combining the blockade of NADPH oxidase with other therapeutic strategies to enhance its impact on atherosclerosis development.

It should be noted that some drugs, like metformin or liraglutide, inhibit hyperglycemia-induced ROS production through the downregulation of PKC-dependent NADPH oxidase in human ECs [19].

**Figure 8:**
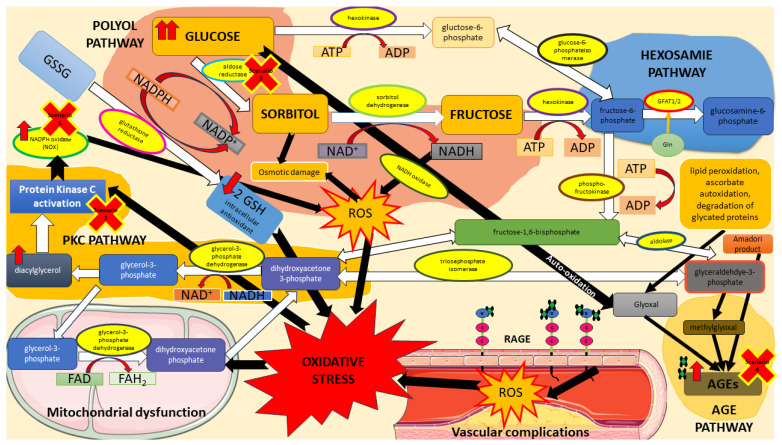
Figure presenting the relationship between glucose flow through the polyol pathway and oxidative stress and their impact on atherosclerosis. Red crosses mark the inhibition sites that reflect the scenarios analyzed in the study. Parts of the figure were created using images from Servier Medical Art. Servier Medical Art by Servier is licensed under a Creative Commons Attribution 3.0 Unported License (https://creativecommons.org/licenses/by/3.0/, accessed on 9 October 2023).

In Figure 8, the scheme of the study has been presented. It is based on the main pathways that are involved in DM-related atherosclerosis. Activation of the polyol pathway contributes to the following: (A) fructose and its metabolites are strong glycating agents, and when their production is accelerated, more AGEs are produced; (B) in the polyol pathway, where NADPH is converted to NADP${}^{+}$ and NAD${}^{+}$ to NADH, NADPH depletion reduces the production of reduced glutathione, which accelerates oxidative stress; (C) an increase in NADH levels causes an increase in glycerol-3-phosphate levels, which activates protein kinase C (PKC pathway). The figure further highlights the scenarios that were considered in this study (except Scenario 1, which was used to identify important pathways), showing possible capture points for inhibiting compounds that could be useful in reducing atherosclerosis underlying DM.

### 3.3. Limitations of the Study

Classical Petri net-based models primarily focus on portraying the system’s structure. While this may seem like a significant limitation, it is particularly relevant in the case of complex biological systems, where the structure is often crucial for the functionality of the modeled system. Therefore, qualitative models can be instrumental in uncovering vital characteristics of such systems. By analyzing the net structure, important system properties and interactions between various subprocesses can be identified, providing valuable insights despite the qualitative nature of the modeling.

Nevertheless, such approaches have their limitations. One of them is that Petri nets represent a simplified view of the underlying biological processes. The model might not include some complex interactions, nonlinear dynamics, and other quantitative factors. Furthermore, simulating knockouts of reactions and processes is a theoretical approach that might not capture all the consequences of actual genetic or pharmacological knockouts in a real biological system (however, the same can be said about models of other types). Other limitations may arise from assumption-based biases and neglecting stochastic effects. Finally, classical Petri nets might not capture the time-dependent behavior of the processes, which is often important in understanding the progression or treatment of diseases like atherosclerosis.

Despite these limitations, the usefulness of qualitative Petri net-based models or knockout-based analysis should not be underestimated or disregarded. Instead, caution must be taken in interpreting the results, and ideally, the predictions made based on the model should be validated experimentally. However, securing exhaustive experimental data for every aspect of a complex, prolonged condition such as atherosclerosis might not always be achievable.

### 3.4. Potential Clinical Implications for Hyperglycemic Patients with Atherosclerosis

The Petri net model has the potential to serve as a diagnostic tool for hyperglycemic patients with atherosclerosis, offering insights into disease progression, treatment pathways, risk assessment, and therapeutic monitoring. However, translating these in silico findings into clinical practice presents challenges. These include ensuring model accuracy, accounting for individual variations, addressing the complexities of biological systems, and the need for rigorous clinical trials. Additionally, there are ethical concerns related to acting on model predictions without empirical evidence and the necessity to educate healthcare professionals about the model’s intricacies. In essence, while the Petri net model holds promise, its real-world clinical application demands careful consideration and a multidisciplinary approach.

## 4. Conclusions

Although hyperglycemia remains the primary goal in the treatment of chronic complications of diabetes, it is now clear that therapies should also address factors indirectly related to glycemia control. Based on the results of the in silico analyses performed in this study, it can be seen that blocking PKC (β and γ) in hyperglycemic patients can effectively slowdown the progression of atherosclerosis.

In addition, it has been confirmed that the blockade of AR can diminish atherosclerosis; additionally, this blockade has been shown to reduce PKC (β and γ) expression in the course of diabetes.

It has also been observed that reducing oxidative stress by focusing on the AGE-RAGE axis may be a promising therapeutic approach in treating atherosclerosis in diabetics.

However, the blockade of NADPH oxidase, the key enzyme responsible for the formation of ROS in human blood vessels, playing a crucial role in the initiation and progression of atherosclerosis, only moderately slowed down the development of atherosclerosis. It should be emphasized here that, unlike AR blockade, or direct PKC (β and γ), the increased production of mitochondrial ROS associated with mitochondrial dysfunction effectively stopped after NADPH oxidase blockade.

The proposed model and the performed analyses are an attempt to further understand the processes of atherosclerosis formation and development. Since these processes are very complex, their investigation using systems approaches seems to be necessary, and Petri nets are one of the promising mathematical tools that can be used for this purpose.

## Figures and Tables

**Table 1:** The MCT sets of the model and their biological interpretations.

MCT Set	Contained Transitions	Biological Interpretation
$m_{1}$	$t_{24}$, $t_{32}$, $t_{33}$, $t_{35}$, $t_{36}$, $t_{37}$, $t_{41}$, $t_{42}$, $t_{44}$, $t_{47}$, $t_{53}$, $t_{54}$, $t_{56}$, $t_{58}$, $t_{60}$, $t_{63}$, $t_{64}$	Protein kinase C pathway.
$m_{2}$	$t_{0}$, $t_{1}$, $t_{2}$, $t_{3}$, $t_{4}$, $t_{5}$, $t_{6}$, $t_{22}$, $t_{48}$, $t_{51}$, $t_{52}$, $t_{59}$, $t_{61}$	Atherosclerosis development and progression affected by ROS and oxidized LDL.
$m_{3}$	$t_{28}$, $t_{30}$, $t_{31}$, $t_{34}$, $t_{65}$, $t_{66}$, $t_{67}$, $t_{68}$, $t_{69}$, $t_{70}$, $t_{71}$, $t_{72}$	Increased production of ROS as a consequence of mitochondrial dysfunction resulting from hyperglycemia.
$m_{4}$	$t_{16}$, $t_{19}$, $t_{21}$	Activation of the thromboxane receptor (TP) leading to apoptosis, vascular muscle cell activation, and vascular cell adhesion molecules expression.
$m_{5}$	$t_{25}$, $t_{26}$, $t_{27}$	Metabolic pathway that converts glucose into glyceraldehyde 3-phosphate.
$m_{6}$	$t_{39}$, $t_{40}$, $t_{49}$	Antioxidant defense mechanism involving glutathione-dependent enzymes.
$m_{7}$	$t_{9}$, $t_{10}$	Impact of peroxynitrite on nitric oxide (NO) synthesis.
$m_{8}$	$t_{12}$, $t_{13}$	Vasoconstriction and endothelial dysfunction induced by PGI${}_{2}$ synthase disruption and inactivation.
$m_{9}$	$t_{43}$, $t_{46}$	Activation of proatherogenic proteins leading to atherosclerosis progression.
$m_{10}$	$t_{73}$, $t_{74}$	Glucose uptake and transport across the cell membrane.

**Table 3:** The effect of eliminating certain transitions that are associated with the progression of atherosclerosis.

Inhibited Process/Molecule	Knocked-Out Transitions, and MCT Sets	Disabled Transitions and MCT Sets	Number of Remaining t-Invariants (Percentage of Remaining t-Invariants)	The Number of Remaining t-Invariants That Include Transition $t_{43}$ (Atherosclerosis Progression), in Their Supports (Percentage of Remaining t-Invariants That Contain Transition $t_{43}$ in Their Supports)
Inhibition of PKC (β and γ) pathway in glucose metabolism (see Scenario 2)	$t_{55}$, $t_{58}$	$m_{1}$, $m_{2}$, $m_{7}$, $m_{9}$, $t_{15}$, $t_{17}$, $t_{38}$, $t_{45}$, $t_{55}$, $t_{57}$, $t_{62}$	14 (out of 150) (9.33%)	0 (out of 90) (0%)
Inhibition of polyol pathway in glucose metabolism (see Scenario 3)	$t_{32}$	$m_{1}$, $m_{2}$, $m_{9}$, $t_{15}$, $t_{17}$, $t_{38}$, $t_{45}$	27 (out of 150) (18.00%)	0 (out of 90) (0%)
Inhibition of the advanced glycation end-products (AGEs) (see Scenario 4)	$t_{63}$	$m_{1}$, $m_{2}$, $m_{9}$, $t_{15}$, $t_{17}$, $t_{38}$, $t_{45}$	27 (out of 150) (18.00%)	0 (out of 90) (0%)
Inhibition of NADPH oxidase (see Scenario 5)	$t_{34}$, $t_{62}$	$m_{3}$	71 (out of 150) (47.33%)	42 (out of 90) (46.66%)
Inhibition of NADPH oxidase and oxidized LDL formation (see Scenario 5)	$t_{0}$, $t_{34}$, $t_{62}$	$m_{2}$, $m_{3}$	47 (out of 150) (31.33%)	18 (out of 90) (20.00%)
Inhibition of NADPH oxidase and peroxynitrite formation (see Scenario 5)	$t_{7}$, $t_{34}$, $t_{62}$	$m_{2}$, $m_{3}$, $m_{4}$, $m_{8}$, $m_{9}$, $t_{8}$, $t_{14}$, $t_{15}$, $t_{17}$, $t_{18}$, $t_{20}$, $t_{57}$	6 (out of 150) (4.00%)	0 (out of 90) (0%)
Inhibition of NADPH oxidase and AGE formation (see Scenario 5)	$t_{34}$, $t_{36}$, $t_{62}$	$m_{1}$, $m_{2}$, $m_{3}$, $m_{5}$, $m_{9}$, $m_{10}$, $t_{15}$, $t_{17}$, $t_{38}$, $t_{45}$, $t_{62}$, $t_{75}$, $t_{76}$	11 (out of 150) (7.33%)	0 (out of 90) (0%)

## Data Availability

The data presented in this study are available in this article.

## Abbreviations

The following abbreviations are used in this manuscript:

AGEs	advanced glycation end products
AKT	protein kinase B
ATP	adenosine triphosphate
BH4	tetrahydrobiopterin
CO${}_{2}$	carbon dioxide
CVD	cardiovascular disease
DAG	diacylglycerol
DHAP	dihydroxyacetone phosphate
DM	diabetes mellitus
ECM	extracellular matrix
ECs	endothelial cells
Egr-1	early growth response protein 1
ELAM-1	endothelial-leukocyte adhesion molecule 1
eNOS	endothelial nitric oxide synthase
ERK 1/2	extracellular signal-regulated kinase 1/2
ET-1	endothelin 1
ETC	electron transport chain
ER	endoplasmic reticulum
FAD	flavin adenine dinucleotide
FADH${}_{2}$	reduced flavin adenine dinucleotide
GAPDH	glyceraldehyde-3-phosphate dehydrogenase
GFAT	catalyzed by glutamine:fructose-6-phosphate aminotransferase
GLUT	glucose transporter
GPx	glutathione peroxidase
GPDH	glycerol-3-phosphate dehydrogenase
GSH	glutathione
GSSG	glutathione disulfide
HDAC3	histone deacetylase 3
ICAM-1	intercellular adhesion molecule 1
JAKs	Janus kinases
LDL	low-density lipoproteins
MAPK	mitogen-activated protein kinase
MCP-1	monocyte chemoattractant protein
MGO	methylglyoxal
MyD88	differentiation factor 88
NAD	nicotinamide adenine dinucleotide
NADH	reduced nicotinamide adenine dinucleotide
NADP	nicotinamide adenine dinucleotide phosphate
NADPH oxidase	nicotinamide adenine dinucleotide phosphate oxidase
NO	nitric oxide
NOX	NADPH oxidase
NF-κB	nuclear factor-κB
PGI${}_{2}$	prostacyclin 2
PGH${}_{2}$	prostaglandin H${}_{2}$
PKC	protein kinase C
PPARγ	peroxisome proliferator-activated receptor γ
RAGE	receptor for advanced glycation end products
RNS	reactive nitrogen species
ROS	reactive oxygen species
SDH	succinate dehydrogenase
Sirt-1	sirtuin 1
STATs	signal transducer and activator of transcription proteins
T1DM	type 1 diabetes mellitus
TCA	tricarboxylic acid
TIR	toll-interleukin-1 receptor
TIRAP	toll-interleukin-1 receptor (TIR) domain-containing adaptor protein
TP	thromboxane receptor
T${}_{x}$A${}_{2}$	thromboxane A2
UDP GlcNAc	uridine 5${}^{\prime }$-diphospho-N-acetylglucosamine
VCAM-1	vascular cell adhesion molecule 1

## Appendix A

**Table A1:** The list of places and their biological meaning.

Place	Biological Meaning	References	Place	Biological Meaning	References
$p_{0}$	oxidized LDL	[7,36]	$p_{33}$	AGE-RAGE complex	[27]
$p_{1}$	LDL	[7,36]	$p_{34}$	glutathione disulfide (GSSG)	[6,25]
$p_{2}$	ROS	[6,15,16,17,19,25,26,27,36]	$p_{35}$	glutathione (GSH)	[6,7,25]
$p_{3}$	monocytes	[7,36]	$p_{36}$	water	[6,25]
$p_{4}$	MCP-1	[7,36]	$p_{37}$	increase glucose conversion into sorbitol	[6,26]
$p_{5}$	macrophages	[7,36]	$p_{38}$	glucosamine-6P	[6,20]
$p_{6}$	ECM	[7,36]	$p_{39}$	increase fructose-6P conversion into glucosamine-6P	[6,26]
$p_{7}$	LDL in the vessel wall	[7,36]	$p_{40}$	glutamine (Gln)	[6,18,19,20]
$p_{8}$	macrophages with scavenger receptors	[7,36]	$p_{41}$	UDP-GlcNAc	[6,20]
$p_{9}$	foam cells	[7,36]	$p_{42}$	proteins O-GlcNAcylated	[6,20]
$p_{10}$	peroxynitrite	[15,16,17]	$p_{43}$	proatherogenic proteins	[6,20]
$p_{11}$	eNOS uncoupled	[15,16,17]	$p_{44}$	atherosclerotic plaque growth	[7,36]
$p_{12}$	NO level decreased	[15,16,17]	$p_{45}$	RAGE receptor	[27]
$p_{13}$	low PGI${}_{2}$	[15,16,17]	$p_{46}$	DHAP	[18,19]
$p_{14}$	PGH${}_{2}$	[15,16,17]	$p_{47}$	increase glyceraldehyde conversion into DHAP	[6,26]
$p_{15}$	thromboxane A${}_{2}$	[6,15,16,17,19]	$p_{48}$	glycerol-3-phosphate	[18,19]
$p_{16}$	TP receptor activated	[6,15,16,17,19]	$p_{49}$	DAG	[6,18,19,25]
$p_{17}$	high PGI${}_{2}$	[15,16,17]	$p_{50}$	PKC (β and γ) activated	[6,19]
$p_{18}$	T${}_{x}$A${}_{2}$ synthase	[6,19]	$p_{51}$	NF-κB	[15,16,17]
$p_{19}$	glucose	[6,11,22]	$p_{52}$	VCAM-1 and ICAM-1	[15,16,17]
$p_{20}$	glucose-6-P	[6,11]	$p_{53}$	MGO	[6,26]
$p_{21}$	fructose-6-P	[6,11,20]	$p_{54}$	increase glyceraldehyde-3-P conversion into MGO	[6,26]
$p_{22}$	glyceraldehyde-3-P	[6,11]	$p_{55}$	AGE	[26,27]
$p_{23}$	NAD	[12,13,22]	$p_{56}$	NF-κB upregulated	[15,16,17]
$p_{24}$	NADP	[6,25]	$p_{57}$	pyruvate	[6,11]
$p_{25}$	1,3-diphospho-glycerate	[6,11]	$p_{58}$	pyruvate in mitochondia	[12,13]
$p_{26}$	GAPDH active	[6,11,20]	$p_{59}$	CO${}_{2}$	[12,13]
$p_{27}$	NADH	[6,12,13,22,25]	$p_{60}$	FAD	[6,11,12,13]
$p_{28}$	sorbitol	[22]	$p_{61}$	FADH2	[6,12,13]
$p_{29}$	NADPH	[6,22,25]	$p_{62}$	ETC complex I	[12,13]
$p_{30}$	fructose	[22]	$p_{63}$	ETC complex II	[12,13]
$p_{31}$	metabolites of fructose	[22]	$p_{64}$	ETC coenzyme Q	[12,13]
$p_{32}$	AGE increased	[27]	$p_{65}$	GLUT (transporter)	[6,11,22]

**Table A2:** The list of transitions and their biological meaning.

Transition	Biological Meaning	References	Transition	Biological Meaning	References
$t_{0}$	oxidation	[7,36]	$t_{39}$	reaction catalyzed by glutathione reductase	[6,25]
$t_{1}$	endothelial cells stimulation	[7,36]	$t_{40}$	reaction catalyzed by glutathione peroxidase GPx	[6,25]
$t_{2}$	attracting monocytes into subendothelial space	[7,36]	$t_{41}$	reaction catalyzed by GFAT	[6,20]
$t_{3}$	LDL and ECM interaction	[7,36]	$t_{42}$	reaction forming UDP-GlcNAc	[6,20]
$t_{4}$	transformation into macrophages	[7,36]	$t_{43}$	atherosclerosis progression	[5,6,7,20,36]
$t_{5}$	scavenger receptors expression	[7,36]	$t_{44}$	proteins O-GlcNAcylation	[6,20]
$t_{6}$	foam cells formation stimulation	[7,36]	$t_{45}$	ubiquitination degradation of atheroprotective proteins	[6,20]
$t_{7}$	ROS reaction with NO	[15,16,17]	$t_{46}$	increased transcription of proatherogenic proteins	[6,20]
$t_{8}$	ONOO interaction with eNOS cofactor BH4	[15,16,17]	$t_{47}$	synthesis by glutamine synthetase	[6,20]
$t_{9}$	decreased NO synthesis	[15,16,17]	$t_{48}$	extracellular molecules secreted by cells	[7,36]
$t_{10}$	ROS production increased through decreased NO	[15,16,17]	$t_{49}$	water usage	[6,25]
$t_{11}$	ROS production via eNOS blocking	[15,16,17]	$t_{50}$	NADPH formation	[6,22,25]
$t_{12}$	PGI${}_{2}$ synthase disrupting	[15,16,17]	$t_{51}$	foam cells accumulation	[7,36]
$t_{13}$	PGH2 buildup	[15,16,17]	$t_{52}$	LDL formation	[7,36]
$t_{14}$	vasoconstriction	[15,16,17]	$t_{53}$	RAGE expression	[27]
$t_{15}$	endothelial dysfunction	[15,16,17]	$t_{54}$	reaction catalyzed by triose phosphate isomerase	[27]
$t_{16}$	PGI${}_{2}$ conversion by TxA2 synthase	[6,15,16,17,19]	$t_{55}$	PKC (β and γ) expression	[6,19,27]
$t_{17}$	vascular smooth muscles activation	[6,15,16,17,19]	$t_{56}$	reaction catalyzed by GPDH	[18,19]
$t_{18}$	apoptosis	[6,15,16,17,19]	$t_{57}$	increase in T${}_{x}$A${}_{2}$	[6,19]
$t_{19}$	TP receptor activation	[6,15,16,17,19]	$t_{58}$	PKC (β and γ) activation by DAG	[18,19]
$t_{20}$	T${}_{x}$A${}_{2}$ synthase expression		$t_{59}$	NF-κB expression	[6,15,16,17]
$t_{21}$	PGI${}_{2}$ synthase active	[15,16,17]	$t_{60}$	reaction forming DAG	[18,19]
$t_{22}$	adhesion molecules (ICAM-1, VCAM-1, ELAM-1) expression	[6,15,16,17]	$t_{61}$	NF-κB upregulation	[6,15,16,17]
$t_{23}$	eNOS coupled	[15,16,17]	$t_{62}$	ROS production by activated NADPH dependent oxidase	[6,19]
$t_{24}$	GAPDH inactive	[6,26]	$t_{63}$	reaction forming MGO	[6,26]
$t_{25}$	reaction catalyzed by hexokinase	[6,11]	$t_{64}$	reaction forming AGE	[26,27]
$t_{26}$	reaction catalyzed by phosphoglucoisomerase	[6,11]	$t_{65}$	reaction forming pyruvate	[6,11]
$t_{27}$	reaction forming glyceraldehyde-3-P	[6,11]	$t_{66}$	pyruvate transported into mitochondria	[6,12,13]
$t_{28}$	NAD formation	[12,13,22]	$t_{67}$	pyruvate enters TCA cycle	[12,13]
$t_{29}$	NADP formation	[22]	$t_{68}$	ROS generated by increased electron donation to coenzyme Q	[12,13]
$t_{30}$	reaction catalyzed by GAPDH	[6,11]	$t_{69}$	CO${}_{2}$ usage	[12,13]
$t_{31}$	GAPDH activation	[6,11]	$t_{70}$	electrons donation to ETC complex I	[12,13]
$t_{32}$	reaction catalyzed by aldose reductase	[22]	$t_{71}$	electrons passed to ETC coenzyme Q	[12,13]
$t_{33}$	oxidation catalyzed by SDH	[22]	$t_{72}$	electrons donation to ETC complex II	[12,13]
$t_{34}$	ROS production via NADPH oxidase by increased NADH	[6,12,13,22,25]	$t_{73}$	GLUT expression	[6,11,22]
$t_{35}$	reaction forming metabolites of fructose	[22]	$t_{74}$	glucose uptake	[6,11,22]
$t_{36}$	increase in AGE formation	[6,25,26,27]	$t_{75}$	food intake	[22]
$t_{37}$	AGE binding to receptor RAGE	[27]	$t_{76}$	gluconeogenesis	[6,11,22]
$t_{38}$	ROS production by AGE-RAGE complex	[27]	$t_{77}$	NADP usage	[22]

## References

[1] Bhatti J., Sehrawat A., Mishra J., Sidhu I., Navik U., Khullar N., Kumar S., Bhatti G., Reddy P. (2022). Oxidative stress in the pathophysiology of type 2 diabetes and related complications: Current therapeutics strategies and future perspectives. Free Radic. Biol. Med..

[2] Sakran N., Graham Y., Pintar T., Yang W., Kassir R., Willigendael E., Singhal R., Kooreman Z., Ramnarain D., Mahawar K. (2022). The many faces of diabetes. Is there a need for re-classification? A narrative review. BMC Endocr. Disord..

[3] Lubawy D., Formanowicz D. (2022). Insulin Resistance and Urolithiasis as a Challenge for a Dietitian. Int. J. Environ. Res. Public Health.

[4] Poznyak A.V., Nikiforov N.G., Markin A.M., Kashirskikh D.A., Myasoedova V.A., Gerasimova E.V., Orekhov A.N. (2021). Overview of OxLDL and Its Impact on Cardiovascular Health: Focus on Atherosclerosis. Front. Pharmacol..

[5] Mudau M., Genis A., Lochner A., Strijdom H. (2012). Endothelial dysfunction: The early predictor of atherosclerosis: Review article. Cardiovasc. J. Afr..

[6] Siracuse J.J., Chaikof E.L., Shrikhande G., McKinsey J. (2012). The Pathogenesis of Diabetic Atherosclerosis. Diabetes and Peripheral Vascular Disease. Contemporary Diabetes.

[7] Jebari-Benslaiman S., Galicia-García U., Larrea-Sebal A., Olaetxea J.R., Alloza I., Vandenbroeck K., Benito-Vicente A., Martín C. (2022). Pathophysiology of Atherosclerosis. Int. J. Mol. Sci..

[8] Klipp E., Liebermeister W., Wierling C., Kowald A., Lehrach H., Herwig R. (2009). Systems Biology: A Textbook.

[9] Koch I., Reisig W., Schreiber F. (2011). Modeling in Systems Biology. The Petri Net Approach.

[10] Formanowicz D., Rybarczyk A., Radom M., Tanaś K., Formanowicz P. (2020). A Stochastic Petri Net-Based Model of the Involvement of Interleukin 18 in Atherosclerosis. Int. J. Mol. Sci..

[11] Chandel N. (2021). Carbohydrate Metabolism. Cold Spring. Harb. Perspect. Biol..

[12] Sivitz W., Yorek M. (2010). Mitochondrial dysfunction in diabetes: From molecular mechanisms to functional significance and therapeutic opportunities. Antioxid. Redox. Signal..

[13] Ciccarelli G., Conte S., Cimmino G., Maiorano P., Morrione A., Giordano A. (2023). Mitochondrial Dysfunction: The Hidden Player in the Pathogenesis of Atherosclerosis?. Int. J. Mol. Sci..

[14] Formanowicz D., Kozak A., Formanowicz P. (2012). A Petri net based model of oxidative stress in atherosclerosis. Found. Comput. Decis. Sci..

[15] Hink U., Oelze M., Kolb P., Bachschmid M., Zou M., Daiber A., Mollnau H., August M., Baldus S., Tsilimingas N. (2003). Role for peroxynitrite in the inhibition of prostacyclin synthase in nitrate tolerance. J. Am. Coll. Cardiol..

[16] De Almeida A., de Oliveira J., da Silva Pontes L., de Souza J., Gonçalves T., Dantas S., de Almeida Feitosa M., Silva A., de Medeiros I. (2022). ROS: Basic Concepts, Sources, Cellular Signaling, and its Implications in Aging Pathways. Oxid. Med. Cell. Longev..

[17] Hinton M., Thliveris J., Hatch G., Dakshinamurti S. (2023). Nitric oxide augments signaling for contraction in hypoxic pulmonary arterial smooth muscle-Implications for hypoxic pulmonary hypertension. Front. Physiol..

[18] Kolczynska K., Loza-Valdes A., Hawro I., Sumara G. (2020). Diacylglycerol-evoked activation of PKC and PKD isoforms in regulation of glucose and lipid metabolism: A review. Lipids Health Dis..

[19] Lien C., Chen S., Tsai M., Lin C. (2021). Potential Role of Protein Kinase C in the Pathophysiology of Diabetes-Associated Atherosclerosis. Front. Pharmacol..

[20] Lee S., Park S., Choi C. (2022). Insulin Resistance: From Mechanisms to Therapeutic Strategies. Diabetes Metab. J..

[21] Fiorentino T.V., Procopio T., Mancuso E., Arcidiacono G.P., Andreozzi F., Arturi F., Sciacqua A., Perticone F., Hribal M.L., Sesti G. (2015). SRT1720 counteracts glucosamine-induced endoplasmic reticulum stress and endothelial dysfunction. Cardiovasc. Res..

[22] Moemen L., Abdel Hamid M., Wahab S., Kenawy M., Abuelela M., Hassanin O., Fouly M., Abdelazeem A., Noweir S., Ismail S. (2020). Role of advanced glycation end products and sorbitol dehydrogenase in the pathogenesis of diabetic retinopathy. Bull. Natl. Res. Cent..

[23] Black H.S. (2022). A Synopsis of the Associations of Oxidative Stress, ROS, and Antioxidants with Diabetes Mellitus. Antioxidants.

[24] Wright E., Scism-Bacon J.L., Glass L.C. (2006). Oxidative stress in type 2 diabetes: The role of fasting and postprandial glycaemia. Int. J. Clin. Pract..

[25] Singh M., Kapoor A., Bhatnagar A. (2021). Physiological and Pathological Roles of Aldose Reductase. Metabolites.

[26] Wetzels S., Wouters K., Schalkwijk C., Vanmierlo T., Hendriks J. (2017). Methylglyoxal-Derived Advanced Glycation Endproducts in Multiple Sclerosis. Int. J. Mol. Sci..

[27] Twarda-Clapa A., Olczak A., Białkowska A., Koziołkiewicz M. (2022). Advanced Glycation End-Products (AGEs): Formation, Chemistry, Classification, Receptors, and Diseases Related to AGEs. Cells.

[28] Taguchi K., Fukami K. (2023). RAGE signaling regulates the progression of diabetic complications. Front. Pharmacol..

[29] Rochette L., Zeller M., Cottin Y., Vergely C. (2014). Diabetes, oxidative stress and therapeutic strategies. Biochim. Biophys. Acta Gen. Subj..

[30] Ma X., Chen Z., Wang L., Wang G., Wang Z., Dong X., Wen B., Zhang Z. (2018). The Pathogenesis of Diabetes Mellitus by Oxidative Stress and Inflammation: Its Inhibition by Berberine. Front. Pharmacol..

[31] Ceriello A., Quagliaro L., Catone B., Pascon R., Piazzola M., Bais B., Marra G., Tonutti L., Taboga C., Motz E. (2002). Role of Hyperglycemia in Nitrotyrosine Postprandial Generation. Diabetes Care.

[32] Giacco F., Brownlee M. (2010). Oxidative stress and diabetic complications. Circ. Res..

[33] Poznyak A., Grechko A., Orekhova V., Khotina V., Ivanova E., Orekhov A. (2020). NADPH Oxidases and Their Role in Atherosclerosis. Biomedicines.

[34] Chew P., Yuen D., Stefanovic N., Pete J., Coughlan M., Jandeleit-Dahm K., Thomas M., Rosenfeldt F., Cooper M., de Haan J. (2010). Antiatherosclerotic and renoprotective effects of ebselen in the diabetic apolipoprotein E/GPx1-double knockout mouse. Diabetes.

[35] Bornfeldt K.E. (2016). Does Elevated Glucose Promote Atherosclerosis? Pros and Cons. Circ. Res..

[36] La Sala L., Prattichizzo F., Ceriello A. (2019). The Link Between Diabetes and Atherosclerosis. Eur. J. Prev. Cardiol..

[37] Orchard T.J., Costacou T., Kretowski A., Nesto R.W. (2006). Type 1 Diabetes and Coronary Artery Disease. Diabetes Care.

[38] Poznyak A., Grechko A.V., Poggio P., Myasoedova V.A., Alfieri V., Orekhov A.N. (2020). The Diabetes Mellitus-Atherosclerosis Connection: The Role of Lipid and Glucose Metabolism and Chronic Inflammation. Int. J. Mol. Sci..

[39] Murata T. (1989). Petri nets: Properties, analysis and applications. Proc. IEEE.

[40] David R., Alla H. (2010). Discrete, Continuous, and Hybrid Petri Nets.

[41] Formanowicz D., Kozak A., Głowacki T., Radom M., Formanowicz P. (2013). Hemojuvelin–hepcidin axis modeled and analyzed using Petri nets. J. Biomed. Inform..

[42] Sackmann A., Heiner M., Koch I. (2006). Application of Petri net based analysis techniques to signal transduction pathway. BMC Bioinform..

[43] Formanowicz D., Rybarczyk A., Radom M., Formanowicz P. (2020). A Role of Inflammation and Immunity in Essential Hypertension—Modeled and Analyzed Using Petri Nets. Int. J. Mol. Sci..

[44] Formanowicz D., Radom M., Rybarczyk A., Tanaś K., Formanowicz P. (2022). Control of Cholesterol Metabolism Using a Systems Approach. Biology.

[45] Radom M., Rybarczyk A., Szawulak B., Andrzejewski H., Chabelski P., Kozak A., Formanowicz P. (2017). Holmes: A graphical tool for development, simulation and analysis of Petri net based models of complex biological systems. Bioinformatics.

[46] Einloft J., Ackermann J., Nöthen J., Koch I. (2013). MonaLisa—Visualization and analysis of functional modules in biochemical networks. Bioinformatics.

[47] Radom M., Szawulak B. (2022). Holmes 1.1. User Manual. http://www.cs.put.poznan.pl/mradom/Holmes/HolmesEN_v1.10.pdf.

[48] Jubaidi F., Zainalabidin S., Taib I., Abdul Hamid Z., Mohamad Anuar N., Jalil J., Mohd Nor N., Budin S. (2022). The Role of PKC-MAPK Signalling Pathways in the Development of Hyperglycemia-Induced Cardiovascular Complications. Int. J. Mol. Sci..

[49] Grunwald S., Speer A., Ackermann J., Koch I. (2008). Petri net modelling of gene regulation of the Duchenne muscular dystrophy. Biosystems.

[50] Pan D., Xu L., Guo M. (2022). The role of protein kinase C in diabetic microvascular complications. Front. Endocrinol..

[51] AlQuadeib B.T., Aleanizy F.S., Alqahtani F.Y., Alshammari R.A., Aldarwesh A., Alsarra I. (2023). Determination of Ruboxistaurin analysis in rat plasma utilizing LC–MS/MS technique. Saudi Pharm. J..

[52] Aruni B., Satish K.S. (1992). Aldose reductase: Congenial and injurious profiles of an enigmatic enzyme. Biochem. Med. Metab. Biol..

[53] Wai H.T., Jeremiah S., Scott G., Concetta D.F., Ettore P., Cristiano F., Stefania T., Marta C., Virgilio E., Giacomo L. (2011). Glucose and collagen regulate human platelet activity through aldose reductase induction of thromboxane. J. Clin. Investig..

[54] Vikramadithyan R.K., Hu Y., Noh H.L., Liang C.P., Hallam K., Tall A.R., Ramasamy R., Goldberg I.J. (2005). Human aldose reductase expression accelerates diabetic atherosclerosis in transgenic mice. J. Clin. Investig..

[55] Jannapureddy S., Sharma M., Yepuri G., Schmidt A.M., Ramasamy R. (2021). Aldose Reductase: An Emerging Target for Development of Interventions for Diabetic Cardiovascular Complications. Front. Endocrinol..

[56] Ramana K., Srivastava S. (2010). Aldose reductase: A novel therapeutic target for inflammatory pathologies. Int. J. Biochem. Cell Biol..

[57] Gopal K., Karwi Q., Tabatabaei Dakhili S., Wagg C., Zhang L., Sun Q., Saed T., Panidarapu S., Perfetti R., Ramasamy R. (2023). Aldose reductase inhibition alleviates diabetic cardiomyopathy and is associated with a decrease in myocardial fatty acid oxidation. Int. J. Biochem. Cell Biol..

[58] Li Q., Wang Z., Zhou W., Fan S., Ma R., Xue L., Yang L., Li Y., Tan H., Shao Q. (2016). Epalrestat protects against diabetic peripheral neuropathy by alleviating oxidative stress and inhibiting polyol pathway. Neural Regen. Res..

[59] Oates P. (2008). Aldose reductase, still a compelling target for diabetic neuropathy. Curr. Drug Targets.

[60] Alexiou P., Pegklidou K., Chatzopoulou M., Nicolaou I., Demopoulos J. (2009). Aldose Reductase Enzyme and its Implication to Major Health Problems of the 21st Century. Curr. Med. Chem..

[61] Sarikaya M., Yazihan N., Das Evcimen N. (2020). Relationship between aldose reductase enzyme and the signaling pathway of protein kinase C in an in vitro diabetic retinopathy model. Can. J. Physiol. Pharmacol..

[62] Bansal S., Burman A., Tripathi A. (2023). Advanced glycation end products: Key mediator and therapeutic target of cardiovascular complications in diabetes. World J. Diabetes.

[63] Testa R., Bonfigli A.R., Prattichizzo F., La Sala L., De Nigris V., Ceriello A. (2017). The “Metabolic Memory” Theory and the Early Treatment of Hyperglycemia in Prevention of Diabetic Complications. Nutrients.

[64] Marinos K., Dimitrios D., Phaedon D.Z., Christina P., Athanasios G.P. (2019). Impact of advanced glycation end products (AGEs) signaling in coronary artery disease. Biochim. Biophys. Acta Mol. Basis Dis..

[65] Yamagishi S., Matsui T. (2018). Role of Hyperglycemia-Induced Advanced Glycation End Product (AGE) Accumulation in Atherosclerosis. Ann. Vasc. Dis..

[66] Oshitari T. (2023). Advanced Glycation End-Products and Diabetic Neuropathy of the Retina. Int. J. Mol. Sci..

[67] Sourris K., Watson A., Jandeleit-Dahm K. (2021). Inhibitors of Advanced Glycation End Product (AGE) Formation and Accumulation. Handb. Exp. Pharmacol..

[68] Samuel R., Rama N., KiranKumar Y., Stephen S., Gonzales N., Jerry L.N. (2000). Evidence that pioglitazone, metformin and pentoxifylline are inhibitors of glycation. Clin. Chim. Acta.

[69] Miyata T., van Ypersele D., Ueda Y., Ichimori K., Inagi R., Onogi H., Ishikawa N., Nangaku M., Kurokawa K. (2002). Angiotensin II receptor antagonists and angiotensin-converting enzyme inhibitors lower in vitro the formation of advanced glycation end products: Biochemical mechanisms. J. Am. Soc. Nephrol..

[70] Li X., Zheng T., Sang S., Lv L. (2014). Quercetin inhibits advanced glycation end product formation by trapping methylglyoxal and glyoxal. J. Agric. Food Chem..

[71] Reynaert N.L., Vanfleteren L.E.G.W., Perkins T.N. (2023). The AGE-RAGE Axis and the Pathophysiology of Multimorbidity in COPD. J. Clin. Med..

[72] Gryszczyńska B., Budzyń M., Formanowicz D., Wanic-Kossowska M., Formanowicz P., Majewski W., Iskra M., Kasprzak M.P. (2020). Selected Atherosclerosis-Related Diseases May Differentially Affect the Relationship between Plasma Advanced Glycation End Products, Receptor sRAGE, and Uric Acid. J. Clin. Med..

[73] Nowotny K., Jung T., Höhn A., Weber D., Grune T. (2015). Advanced Glycation End Products and Oxidative Stress in Type 2 Diabetes Mellitus. Biomolecules.

